# Analysis of water, sanitation, and hygiene facilities using the WASH-FIT approach and its relation to patient satisfaction and maternal mortality at hospitals in Indonesia

**DOI:** 10.3389/fpubh.2024.1322470

**Published:** 2024-02-01

**Authors:** Rina Purwandari, D. Daniel, Firdaus Hafidz

**Affiliations:** ^1^Master of Public Health, Faculty of Medicine, Public Health and Nursing, Universitas Gadjah Mada, Yogyakarta, Indonesia; ^2^Department of Health Behavior, Environment, and Social Medicine, Faculty of Medicine, Public Health and Nursing, Universitas Gadjah Mada, Yogyakarta, Indonesia; ^3^Department of Health Policy and Management, Faculty of Medicine, Public Health and Nursing, Universitas Gadjah Mada, Yogyakarta, Indonesia

**Keywords:** hospital, WASH-FIT, satisfaction, maternal mortality, Indonesia

## Abstract

**Introduction:**

The provision of Water, Sanitation, and Hygiene (WASH) is critical to reducing infection and enhancing the quality of health care services. The study aims to assess WASH facilities in Indonesian hospitals using the Water, Sanitation, and Hygiene Facility Improvement Tool (WASH-FIT) approach and examine their association with customer satisfaction and maternal mortality owing to infection.

**Methods:**

We utilized the national scale Health Facilities Research dataset in Indonesian hospitals in 2019. WASH status is determined using WASH-FIT indicators, i.e., water, sanitation, waste management, hand hygiene, environmental cleaning, and management services, and then divided into three levels: poor, adequate, and good categories.

**Results:**

The majority of hospitals in Indonesia had a good category, i.e., the range of hospitals with a good category was 79–97% nationally, in 6 aspects: water, sanitation, hand hygiene, environmental cleaning, and management services, except for waste management services (13%). Good WASH service facilities are more frequently found in government hospitals than in private and specialized hospitals, while lower-level hospitals tend to have poor levels of all WASH-FIT indicators. There are significant relationships between adequate sanitation services (*β* = 0.724), adequate and good categories of hand hygiene services (*β* = 0.712 and 0.866, respectively), environmental cleaning (*β* = −0.501 and –0.503, respectively), and management (*β* = −0.645 and 0.446, respectively), with the proportion of maternal mortality owing to infection. Furthermore, there was no relationship between WASH-FIT indicators and patient satisfaction, except for good hand hygiene services (*β* = 0.453).

**Discussion:**

Despite good conditions in almost all WASH-FIT indicators, the improvement of waste management is urgently needed to improve the WASH services in hospitals in Indonesia, as also found in other developing countries.

## 1 Introduction

To achieve universal health coverage, the World Health Organization (WHO), The United Nations Children’s Fund (UNICEF), and global health partners have committed that by 2030, every health facility, including water, sanitation, and hygiene (WASH) facilities, must be managed securely (1). The WHO report indicates the 2021 status of WASH facilities in Health Care Facilities (HCF): only about 50%, 78%, and 21% of HCFs had basic hygiene, water, and sanitation services, respectively (2). Insufficient resources and weak monitoring systems are often the reasons for this (3, 4). WHO and UNICEF target to have at least 80% of health service facilities have basic-level WASH services in 2025 (5).

Insufficient access to WASH contributes to an unsanitary environment and the transmission of infection (6), as well as *E. coli* contamination of water sources (7). Inadequate WASH access results in the presence of antimicrobial resistance (AMR) in the environment (8). Poor sanitation and hygiene conditions might also increase the risk of communicable diseases, such as intestinal parasite infections, which can exacerbate pregnancy burdens, such as malnutrition and iron deficiency anemia, for both mother and fetus (9). Sepsis incidents also may occur in poor WASH conditions in HCF (10). Moreover, the survival rate of women who suffer from sepsis is lower than that of mothers suffering from other medical conditions (11).

Furthermore, COVID-19 teach us the importance of adequate WASH services at HCF. More than a quarter of Zimbabwe’s health institutions lack adequate WASH services, increasing the risk of transmission of COVID-19 and other infectious diseases (12). The highest rate of COVID-19 cases was reported in locations with inadequate levels of WASH services and infection prevention vigilance (13).

Adequate provision of WASH services is not only related to the transmission of infection but also the people’s decision to come and be hospitalized in the HCF. A systematic review found that poor WASH conditions in HCF result in patient satisfaction and preference of women to give birth at home, which poses a danger to the health of mothers and newborns (14). Thus, inadequate WASH service in HCF is directly and indirectly related to the patient and mother’s health.

Considering the importance of adequate WASH services in HCF, the Joint Monitoring Program (JMP) WHO-UNICEF monitors and publishes WASH provision conditions based on SDGs indicators, called Water, Sanitation, and Hygiene Facility Improvement Tool (WASH-FIT) (1). The WASH-FIT, i.e., Water and Sanitation for Healthy Facility Improvement Tool, is a risk-based management approach to improve the quality of HCF by assessing seven aspects: water, sanitation, hand hygiene, waste management, environmental cleaning and disinfection, energy and power, and management (15). Previous studies have reported the conditions of the HCF in some developing countries using the WASH-FIT approach, including evaluation of WASH status from a treatment center for COVID-19 in Ghana, COVID-19 isolation facilities in Zimbabwe, and a cross-sectional study in rural health facilities of Pakistan (12, 16, 17).

Despite the improvement in access to adequate WASH services in Indonesia in recent years, there is still a challenge in improving the WASH services at HCF in Indonesia. A previous study using the national data in 2010–2011 estimated that One-fourth of HCF in Indonesia did not have access to basic water and sanitation services and more than two-thirds of HCF lacked handwashing facilities with soap (4). Furthermore, The UNICEF report in 2020 shows that only 30% of Indonesian HCF provided safe water and sanitation services (18). Based on the previous explanation, we may relate this to the high maternal death in hospitals in Indonesia, i.e., 77% (19).

To the best of our knowledge, no study comprehensively analyses the WASH services at HCF in Indonesia using the risk-based management approach, i.e., WASH-FIT. Furthermore, no study investigates the association between WASH services status at HCF and customer satisfaction and maternal mortality at HCF in Indonesia. This study aims to fill that gap. The results can be used by the government to improve the general conditions of WASH services at HCF in Indonesia, e.g., by prioritizing which WASH-FIT aspect should be improved.

## 2 Method

### 2.1 Data source and variables

We utilized the data of the 2019 Health Facility Research (*Rifaskes* in Bahasa) evaluation in Indonesia. The Rifaskes is a cross-sectional national survey of health facilities that has been conducted in all districts and cities in Indonesia since 2011. However, there is no information in the Rifaskes report about how the HCF was selected in each city. The Rifaskes samples are hospitals, health centers, independently practicing physicians, independently practicing midwives, clinics, pharmacies, and health laboratories. In this study, only hospital data were processed. A total of 532 hospital samples across Indonesia were collected through the systematic random sampling method representing about 2,813 hospitals in Indonesia, as of July 2018 (20). The study was approved by the Gadjah Mada University Research Ethics Commission (no. KE/FK/0395/EC/2023).

Based on WASH-FIT, water service, sanitation services, waste management services, hand hygiene services, environmental cleaning services, and management services facilities consist of several essential and advanced indicators. In this study, WASH service facilities were measured based on service availability for each indicator contained in the Rifaskes data as a proxy indicator for WASH-FIT indicators. The WASH-FIT indicators can be locally adapted based on regional or national priorities. Thus, not all indicators in the WASH-FIT are available in the Rifaskes data. The variables used in this study and the variable comparison are presented in Table 1 and Supplementary materials, respectively.

**Table 1:** WASH-FIT variables in the Rifaskes data.

No.	Indicator	Criteria based on Rifaskes
*Water*		
1	Essential	Availability of clean water sources: Local Water Supply Utility
2		Availability of clean water for 24 h
3		Adequate availability of clean water
4		Adequacy of clean water in the emergency room
5	Advanced	Availability of a water reservoir (storage)
*Sanitation*		
1	Essential	Availability of outpatient toilets
2		Availability of staff toilets (in the emergency room)
3		Availability of visitor toilets (in the emergency room)
4	Advanced	Availability of Wastewater Treatment Plant (WTP)
5		Availability of wastewater treatment permit
*Waste management*		
1	Essential	Availability of a separate Hospital Waste Management Unit/Section/Installation
2		Sorting of medical solid waste
3		Sorting method used
4		Methods of infection control carried out in hospitals: pedal bin
5		Methods of treatment of solid medical waste (treatment options) Incineration with incinerators Using an autoclave Using a microwave Buried in the ground with encapsulation techniques Disinfect with disinfectant Burned Treatment of solid medical waste with an incinerator is carried out at this health facility
6		Safety box ownership
7		Needle destroyer ownership
1	Advanced	Storage of radioactive waste in separate containers
2		Storage of cytotoxic waste in separate containers
3		Storage of chemical and pharmaceutical waste in separate containers
4		Availability of temporary storage area for toxic and hazardous waste
5		Methods of infection control carried out in hospitals safety box auto disposable syringe
6		Availability of standard operating procedures (SOP) for waste disposal
7		Methods of infection control carried out in hospitals: Disposable latex gloves
*Hand hygiene*		
1	Essential	Methods of infection control carried out in hospitals • Clean running water • Alcohol handrub
2		Installation of health banners/banners/posters
*Environmental cleaning*		
1	Essential	Existence of SOPs for the usage of personal protective equipment (PPE)
2		Availability of procedures for handling toxic and hazardous waste contamination
3	Advanced	Availability of laundry / laundry services
*Management*		
1	Essential	Availability of hospital strategic plan documents
2		Organizing in carrying out the strategic plan
3		Availability of implementation documents
4		Implementation of evaluation monitoring
5		Availability of budget for the implementation of health promotion activities in hospitals
6		Hospital organizational structure
1	Advanced	Hospital occupational health and safety program (policy)
2		Availability of standard infection prevention precautions guidelines
3		Staff education and training program in occupational safety, fire hazard and disaster in 2018
4		Availability of Nosocomial Infection Control Committee or Infection Prevention and Control (IPC)

### 2.2 Data analysis

WASH status was assessed using six WASH-FIT services, namely the availability of water services, sanitation and waste management services, hand hygiene and environmental hygiene services, and WASH management. The table in the Supplementary File shows the proxy indicators for each service. To estimate the percentage of achievement, the total score for each indication was calculated and divided by the total score of the particular indicator. Based on the percentage, the assessment results were divided into three levels: poor (50%), adequate (50–75%), and good (>75%) (12).

Hospital ownership was categorized into government and private hospitals. Hospital level was categorized into level A (the highest level), level B, level C, level D, and level D-pratama (the lowest level). The types of hospitals were categorized into general hospitals and specialized hospitals, i.e., heart hospitals, eye hospitals, etc. Finally, hospital geographical areas were used as the control variables. The proportion of maternal deaths due to sepsis was calculated from the number of maternal deaths caused by sepsis per number of maternal births in that year. The proportion of patient satisfaction is the percentage of patients who feel satisfied as a result of a satisfaction survey conducted by the hospital that year.

First, the chi-square test was conducted to assess the potential association between the level of WASH service and the characteristics of the hospital, i.e., region, hospital ownership, hospital level, and hospital type. Afterwards, the regression analysis between WASH services and the proportion of maternal mortality due to infection and patient satisfaction was examined using linear regression. The independent variable “WASH status” was treated as the composite variables of water, sanitation, hygiene, and WASH management. Each of the independent variables has three levels of categorical data, namely poor, adequate, and good. The poor category turns into the reference category. The proportion of maternal mortality due to infection and patient satisfaction were the dependent variables, i.e., thus, there are two regressions were conducted.

## 3 Results

### 3.1 Descriptive statistics of the data

The mean value of the proportion of maternal deaths due to sepsis and patient satisfaction is shown in Table 2. The average number of maternities from the 532 HCFs whose data were used in this study in 2018 was 1,023 cases, the average proportion of maternal deaths due to sepsis was 0.58%, and the average proportion of patient satisfaction was 83.04%.

**Table 2:** Maternal mortality and patient satisfaction data.

Variable	Average	SD	Min	Max
Number of maternities in 532 HCFs in 2018	1023.41	1111.23	1	11,827
Proportion of maternal death due to sepsis (%)	0.58	0.97	0	15
Proportion of patient satisfaction levels based on the results of the most recent satisfaction survey (%)	83.04	10.19	0	100

According to Table 3, water, sanitation, hand hygiene, environmental service facilities, as well as management facilities in hospitals, are mostly in the good category. The variable with the highest percentages of “good” status is water services (97.37%), followed by hand hygiene services (90.60%), while only 13% of the HCFs are categorized as “good” waste management services. In general, the conditions of water, sanitation, and hand hygiene services are relatively better than waste management, environmental cleaning, and management services.

**Table 3:** Status of WASH service facility based on WASH-FIT.

Variable	Status	Amount (n)	Percentage (%)
Water service	Poor	0	0
	Adequate	14	2.63
	Good	518	97.37
Sanitation services	Poor	15	2.82
	Adequate	70	13.16
	Good	447	84.02
Waste management services	Poor	41	7.71
	Adequate	418	78.57
	Good	73	13.72
Hand hygiene service	Poor	6	1.13
	Adequate	44	8.27
	Good	482	90.60
Environmental cleaning service	Poor	8	1.50
	Adequate	102	19.17
	Good	422	79.32
Management services	Poor	28	5.26
	Adequate	75	14.10
	Good	429	80.64

### 3.2 The proportion of WASH services-based WASH-FIT

**Figure 1:**
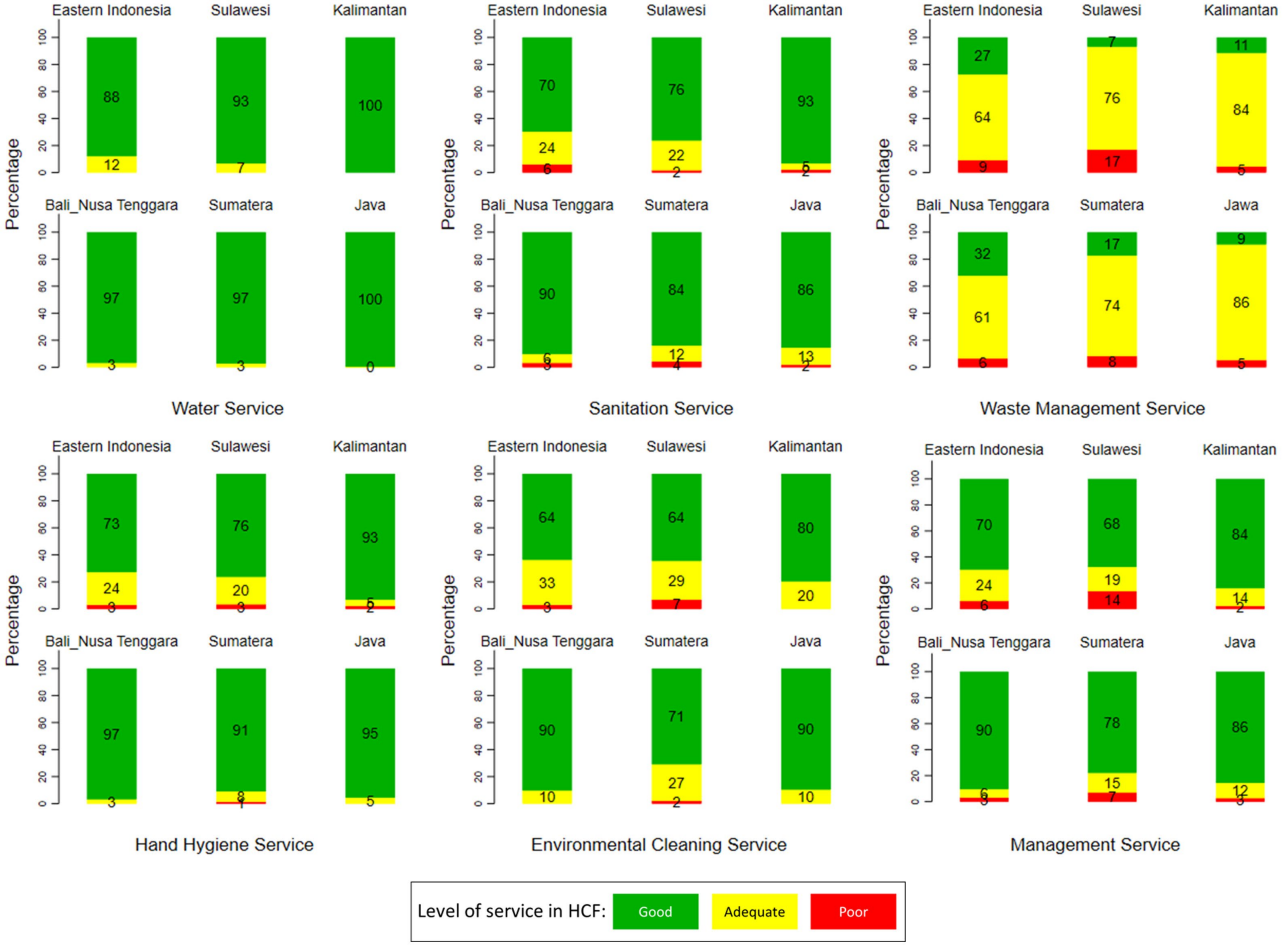
Variation of WASH services at HCF in Indonesia by region (source: The 2019 Health Facility Research).

Figure 1 shows the variation of WASH services by region. Hospitals that had adequate water services, waste management, hand hygiene, environmental cleanliness, and management services were still common in Eastern Indonesia and Sulawesi. On Java Island, there were several hospitals with good water services, waste management, hand hygiene, environmental cleanliness, and management services. Furthermore, adequate condition of wash services seems evenly distributed in all areas.

**Figure 2:**
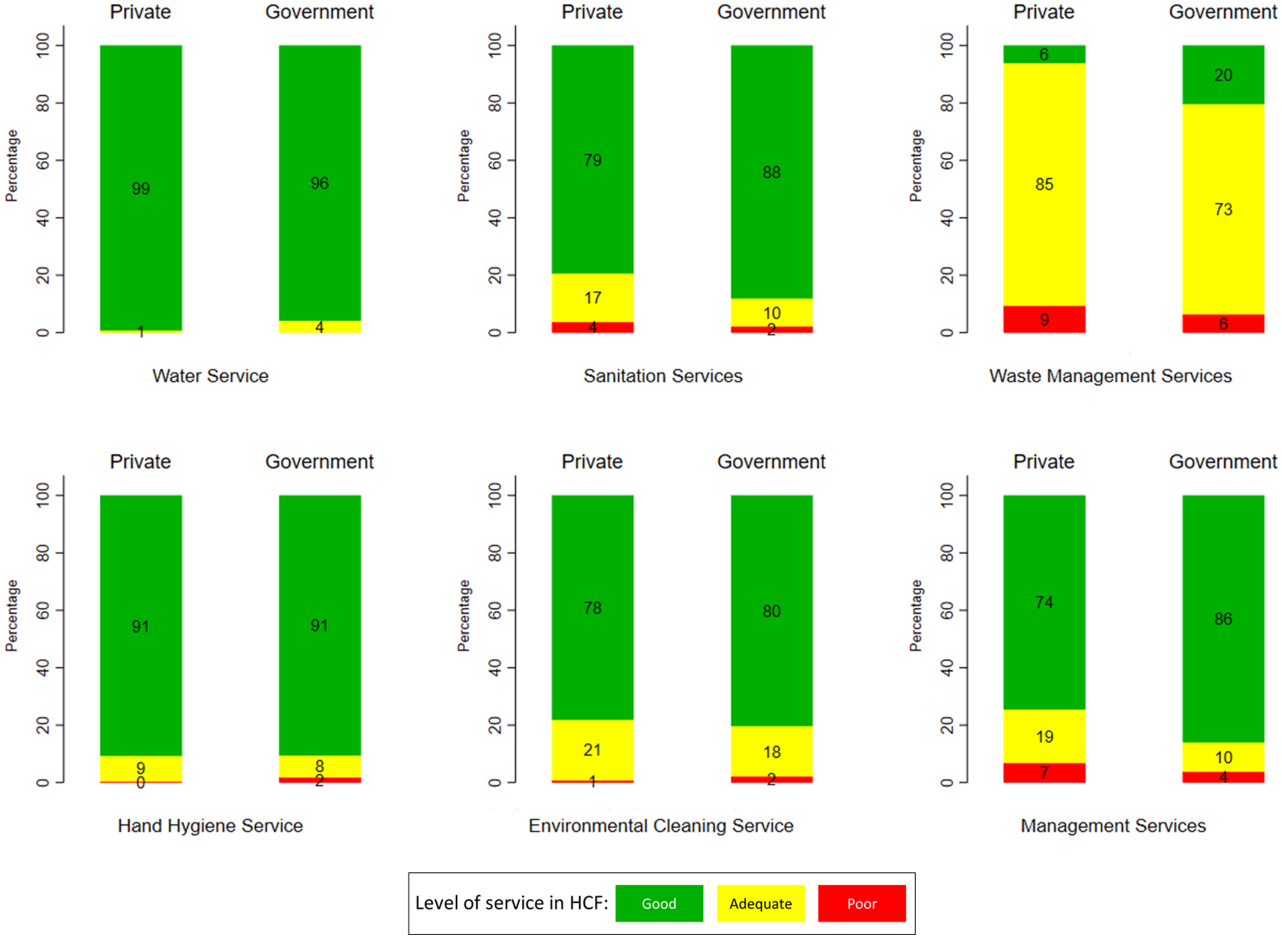
Variation of WASH services at HCF in Indonesia by hospital ownership (source: The 2019 Health Facility Research).

The variation of WASH services by hospital ownership is shown in Figure 2. In general, the conditions of water services, sanitation, waste management, and management services in government hospitals were better than in private hospitals (*p*-value < 0.05). Figure 2 shows that the water service was better than others, regardless of the type of hospital ownership. A prominent difference between government and private hospitals is in the waste management services, i.e., a better condition in government hospitals.

**Figure 3:**
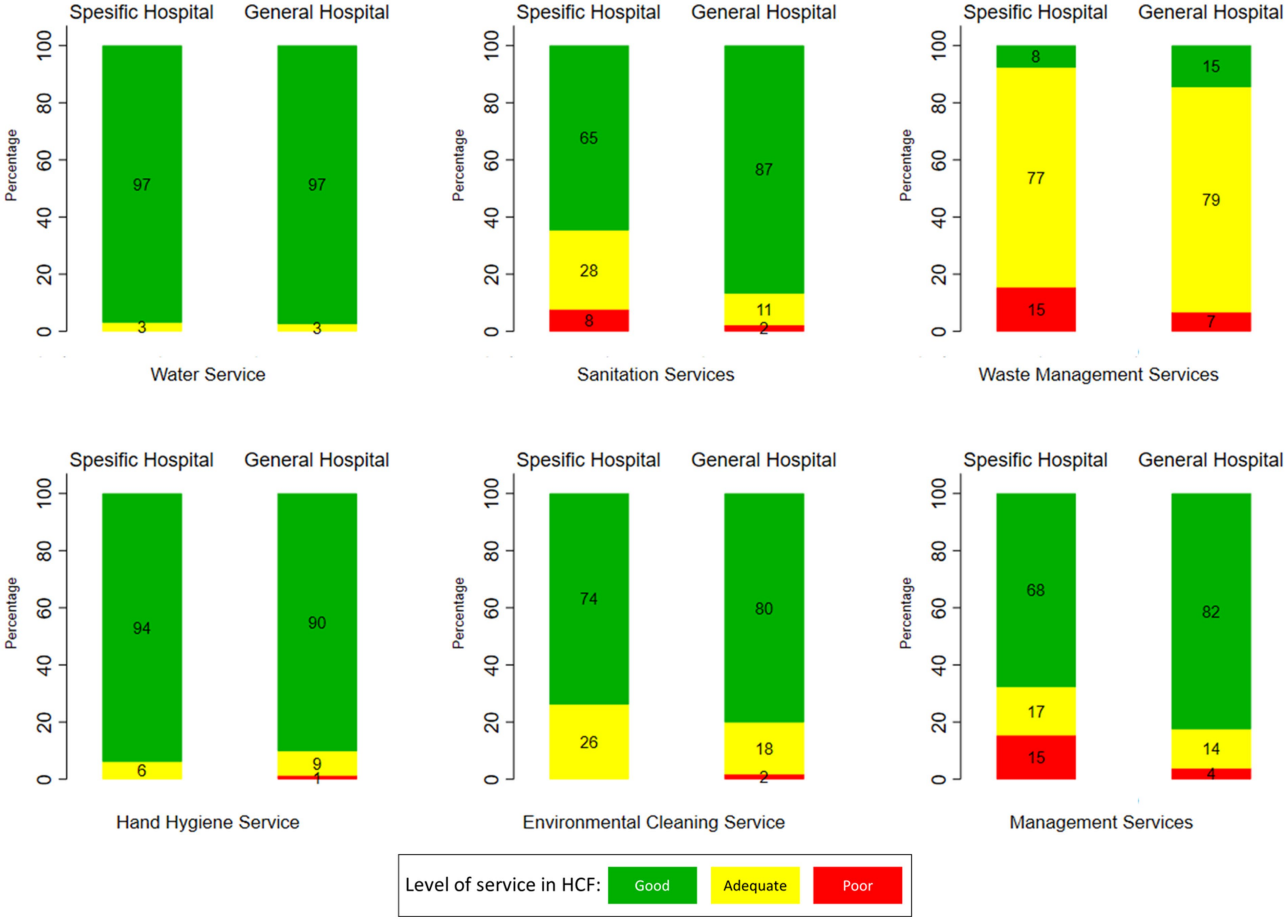
Variation of WASH services at HCF in Indonesia by hospital type (source: The 2019 Health Facility Research).

Figure 3 shows the variation of WASH services by hospital type, i.e., specific or general hospitals. According to Figure 3, hospitals that have good sanitation service facilities are more common in general hospitals (87%) than in specific hospitals (65%). Other prominent differences between these two hospital types are in the waste management and management services, i.e., better conditions in general hospitals.

**Figure 4:**
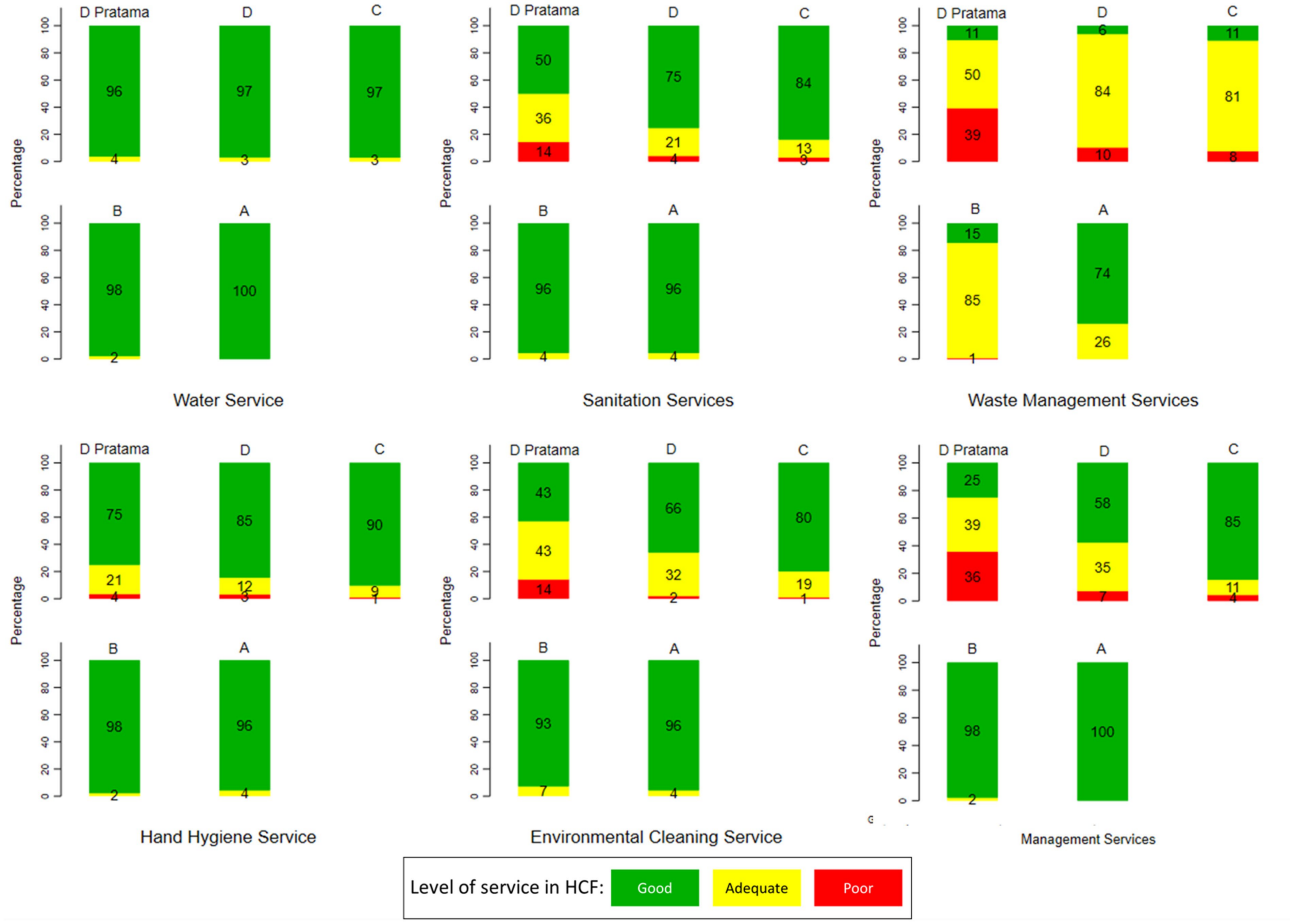
Variation of WASH services at HCF in Indonesia by hospital level. D Pratama is the lowest level of the hospital, while A is the highest level (source: The 2019 Health Facility Research).

Figure 4 shows the variation of WASH services by hospital level. The figures indicate the lower the level of the hospital, e.g., level D Pratama or D, the worse the conditions of the WASH services. In terms of waste management services, most hospitals had an adequate category, except the level A hospitals, which were dominated by a good category of waste management services. Furthermore, almost all levels of the hospital had good water services. However, only sanitation services, waste management, hand hygiene, environmental hygiene, and management services had a significant correlation with hospital class.

Most hospitals, from level D pratama to level A, had good category water service. In terms of sanitation services, there were still hospitals in level C/III, level D/IV, and level D Primary hospitals with poor sanitation services. In terms of waste management services, most hospitals had an adequate category, except level A hospitals, which were dominated by hospitals with a good category of waste management services. Furthermore, in level D hospitals, there were still hospitals with adequate facilities for sanitation (36%), waste management (50%), hand hygiene (21%), environmental cleaning (43%), and management (39%).

### 3.3 Statistical analyses

Chi-square tests found that waste management service was associated with all hospital characteristics (Table 4). Furthermore, region and hospital type were associated with four WASH statuses. Hospital level was associated only with three WASH statuses.

**Table 4:** Result for the Chi-square test between WASH status and hospital characteristics.

WASH status	Hospital characteristics			
	Region	Hospital ownership	Hospital type	Hospital level
Water service	0.001^**^	0.015^*^	0.918	0.811
Sanitation service	0.113	0.024^*^	0.000^***^	0.000^***^
Waste management service	0.000^***^	0.000^***^	0.000^***^	0.022^*^
Hand hygiene service	0.000^***^	0.307	0.003^**^	0.516
Environmental cleaning service	0.000^***^	0.300	0.000^***^	0.194
Management service	0.023^*^	0.004^**^	0.000^***^	0.000^***^

*p-value < 0.001, **p-value < 0.01, ***p-value ≤ 0.05.

The regression found that there are significant relationships between (1) “adequate” sanitation service, (2) “adequate” and “good” hand hygiene services, environmental hygiene, and management services, and (3) hospital level with the proportion of maternal mortality (Table 5). Among all WASH-FIT indicators, the most influential variable associated with the proportion of maternal mortality is a “good” level of hand hygiene services, followed by “adequate” sanitation. Those two variables have a positive association with the proportion of maternal mortality, considering “poor” condition as a reference category.

**Table 5:** A regression linear between WASH services and maternal mortality due to sepsis and patient satisfaction.

Variable		Maternity death^a^			Patient satisfaction^b^		
		*B*	SEB	β	*B*	SEB	β
Water service	Adequate (Ref)	0			0		
	Good	0.561	1.246	0.120	0.104	2.878	0.010
Sanitation services	Poor (Ref)	0			0		
	Adequate	3.389	1.313	0.724**	−0.085	3.032	−0.008
	Good	1.804	1.284	0.385	0.579	2.966	0.057
Waste management services	Poor (Ref)	0			0		
	Adequate	−0.702	0.888	−0.150	−0.331	2.051	−0.032
	Good	−1.162	1.053	−0.248	−0.728	2.431	−0.071
Hand hygiene service	Poor (Ref)	0			0		
	Adequate	3.332	1.97	0.712*	−3.805	4.549	−0.373
	Good	4.055	1.921	0.866**	0.183	4.436	0.018
Environmental cleaning service	Poor (Ref)	0			0		
	Adequate	−2.348	1.048	−0.501**	4.621	2.419	0.453*
	Good	−2.354	1.049	−0.503**	3.178	2.422	0.312
Management services	Poor (Ref)	0			0		
	Adequate	−3.064	1.126	−0.654***	−3.631	2.6	−0.356
	Good	−2.087	1.196	−0.446*	−2.762	2.761	−0.271
Region	Eastern Indonesia (Ref)	0			0		
	Sulawesi	−0.725	0.992	−0.155	0.894	2.29	0.088
	Kalimantan	−0.806	1.058	−0.172	−2.864	2.443	−0.281
	Bali:Nusa Tenggara	−0.125	1.139	−0.027	1.369	2.63	0.134
	Sumatra	−1.044	0.89	−0.223	−0.453	2054	−0.044
	Jawa	−0.729	0.907	−0.156	−0.429	2.094	−0.042
RS type	Special (Ref)	0			0		
	General	0.627	0.672	0.134	0.452	1.552	0.044
Hospital ownership	Private (Ref)	0					
	Government	0.518	0.478	0.111	−2.847	1.103	−0.279**
RS level	D Primary (Ref)	0			0		
	D/IV	−3.61	1.004	−0.771***	−1.117	2.318	−0.110
	C/III	−4.923	0.997	−1.051***	−0.379	2.303	−0.037
	B/II	−4.939	1.089	−1.055***	1.288	2.514	0.126
	A/I	−4.679	1.47	−0.999***	2.06	3.394	0.202

***p-value < 0.01, **p-value < 0.05, *p-value < 0.1. ^a^*R*^2^ = 0.155, ^b^*R*^2^ = 0.049, Ref, reference category.

Furthermore, a significant WASH-FIT variable associated with patient satisfaction is only “adequate” environmental cleaning service. This variable has a positive association with the proportion of patient satisfaction, compared to “poor” condition as a reference category. Outside WASH-FIT variables, government hospital is negatively associated with patient satisfaction. Furthermore, the low percentage variance explained by the model indicates that WASH services are not one of the determinants of patient satisfaction, but other aspects outside WASH-FIT indicators.

## 4 Discussion

### 4.1 Comparison of Rifaskes and WASH-FIT assessment approach

The WASH-FIT assessment can be used to prioritize the improvement of water, sanitation, hygiene, and waste management services at healthcare facilities in low-and middle-income countries, therefore reducing the burden of infectious diseases. Several essential WASH-FIT indicators, such as the availability of separate toilets for men, women, and people with disabilities, as well as menstrual hygiene management facilities for sanitation services, can be adopted in addition to the Rifaskes proxy indicators. Similarly, essential parameters for hand hygiene services, such as the provision of hand washing facilities and informative hand hygiene promotional materials, as well as essential indicators for environmental cleanliness, such as clean room, wall, and floor conditions, and sufficient room lighting, are required (1).

Although a limited number of WASH indicators based on WASH FIT can be measured using Rifaskes data, most of the proxy indicators from Rifaskes can provide an overview of WASH status in hospitals in Indonesia, except hand hygiene and environmental hygiene services. Hand hygiene criteria based on WASH FIT, such as the presence of functional hand washing facilities or sinks and instructions or posters on how to wash hands appropriately, can be integrated for future national research. Assessment criteria for environmental cleanliness indicators include the physical condition of the room, lighting, floor condition, and appropriate and well-maintained materials for cleaning (i.e., detergent, mops, buckets, etc.) are available in the room.

### 4.2 WASH service conditions in HCF in Indonesia based on WASH-FIT assessment

WHO targets to have at least 75% of the HCF in the “good” category, which means that HCF in Indonesia has met the target, except for waste management services, i.e., 13.72%. The conditions in Indonesia are relatively better than in a study reported in Zimbabwe, i.e., <75% of HCFs did not have adequate WASH services (12). Furthermore, the situation of mostly “adequate” level of waste management service in Indonesia is also found in India (17). Also, another study highlights that there is a geographical inequality in medical waste management in Indonesia, in which the medical waste disposal and separation systems in urban and main islands, e.g., Java island, are better than in rural areas (21). This may reflect the imbalance progress of development in Indonesia (22), i.e., between developed or main islands and other islands.

Training and education for staff on standard waste management procedures and government regulations may assist in narrowing the gap in healthcare waste management capabilities, such as observations in Turkey suggesting the success of the government program in reducing the amount of medical waste by sterilizing waste and prohibiting the dumping of medical waste to landfills (23). Training and education about the importance of waste management and the dangers of incompatible waste disposal, such as the emergence of diseases due to infection and the development of insects and animals as disease vectors, has been shown to improve staff understanding of safe medical waste management practices in Bangladesh health facilities (24).

Among all WASH-FIT indicators, water service has the best condition, followed by hand hygiene and sanitation services. This could be because water, sanitation, and hygiene (WASH) aspects are one of the top priorities of the national government, as stated in the national medium-term development plan (“RPJMN” in Bahasa) from 2020 to 2024. Due to this national plan, there has been much improvement in the WASH sector, including in public spaces, like HCF. Water supply services in healthcare facilities are crucial for improving hygiene and sanitation practices and enabling health personnel to implement infection prevention and disease control procedures (25).

### 4.3 Variation of WASH conditions in HFCs in Indonesia based on hospital characteristics

Furthermore, WASH-FIT services are relatively better in government-owned hospitals than in private hospitals. These findings differ from those of Meshi’s study, which indicated that private hospitals have more hand hygiene facilities and waste management guidelines available than government hospitals (25). According to Wijayanti’s study (2015), the increasing number of government hospitals implementing a semi-autonomous financial system (BLU in *Bahasa*) is one of the factors in increasing financial performance in government hospitals. Public service agencies are institutions that provide services to the public while operating on the principle of efficiency and productivity (26). It is possible that implementing the BLU financial system may lead to the improvement of WASH-FIT services.

We also found that government hospitals provided better management services than private hospitals, i.e., 20% compared to 6%. We may argue that better management services in government hospitals than in private hospitals lead to better WASH implementation and conditions. A study by Isunju (27) argues that the monitoring and surveillance system is more difficult to set up in private hospitals than in government hospitals. The difficulties in developing a monitoring and supervision system in private hospitals may be related to the hospital’s leadership style (28). Leaders in private hospitals tend to trust more their subordinates’ performance than in government hospitals. This leads to less strict supervision and monitoring of the hospital’s leader, which is in contrast to the government hospitals. Furthermore, better human resources in government hospitals can also be another reason for this, since private hospital tends to minimize their employees to maximize economic benefits. Government-owned hospitals, as health facilities overseen by the Ministry of Health and the community, must follow the Ministry’s directions and rules. Private hospitals, on the other hand, rely on strategic plans to stay in business (29). Another possible reason is fewer capacity building and training in private hospitals. However, we need more investigation to confirm our argument. All these point out the importance of institutional strengthening for better conditions of WASH services at hospitals, since the institutional aspect, e.g., managerial, monitoring, supervision, leadership, etc., is the key to better WASH services in any setting (30).

Additionally, the conditions of WASH-FIT indicators are relatively better in general hospitals than in specific hospitals. Specific hospitals provide primary care services for a single disease, such as specific hospitals for eye or heart diseases, whereas general hospitals serve all varieties of diseases (31). The reason for this may be that general hospitals provide more health services than specific hospitals, which then increases the income and budget spent on managing service facilities at public hospitals, including WASH service facilities.

Better conditions of WASH-FIT in a higher level of hospitals are in line with our hypothesis. Higher levels of hospitals have higher equipment, number of beds, buildings and infrastructure, human resources, and medical services (32). In addition, government funding support also plays a role in improving WASH facilities in higher levels of hospitals (33).

### 4.4 Relationship analysis of WASH conditions in HCFs in Indonesia and the proportion of maternal mortality due to sepsis and patient satisfaction

The proportion of maternal mortality due to sepsis is significantly correlated with sanitation services, hand hygiene services, environmental hygiene, management services, and hospital level. Among those indicators, the most influential variable associated with the proportion of maternal mortality is good hand hygiene services and adequate sanitation. Some results contradict our hypothesis: better sanitation and hand hygiene services are associated with higher maternal mortality, i.e., the “poor” condition as the reference category.

A positive association between “adequate” sanitation and the proportion of maternal mortality opposed another study that found pregnant women who have access to a toilet have a lower risk of a poor pregnancy outcome, i.e., a negative association (34). However, this result should be interpreted with caution. In the Rifaskes dataset, the sanitation-related variables are “Availability of outpatient toilets” and “Availability of staff toilets (in the emergency room).” These two variables may not fully describe the safely managed sanitation services in the HCF. Furthermore, the availability of “adequate” outpatient toilets and staff toilets may not be enough to fully protect against the spread of pathogens.

Another contradicting finding is a positive association between “adequate” and “good” hand hygiene and the number of maternal mortalities. We may reason that hand hygiene facilities in HCF may not be able to fully protect against the spread of diseases if the HCF’s visitors and health workers do not use it properly and frequently, as suggested by Buxton et al. (35). Another study concluded that the availability of handwashing facilities had no significant effect on adverse pregnancy outcomes in India (34). A study in Cambodia found that only 18% of delivery attendants there adhered to hand hygiene measures before performing labor operations, during delivery, and postpartum (36). By saying this, we do not want to say that a good handwashing facility is not required in HCF, rather arguing that the hygiene practice of the HCF’s visitors and health workers is more important, but this practice is not assessed in Rifaskes dataset. However, our argument still needs further clarification.

Better management and environmental cleaning services are negatively associated with the proportion of maternal mortality. Isunju et al. argue that disease control in HCF is compromised by a variety of factors, including the service management system and a lack of budget (27). A better management system may be characterized by adequate cleaning staff training, which is found in another study related to the number of infections from childbirth in healthcare settings (37). Furthermore, good environmental cleaning services can decrease the potential risks of pathogen exposure on surgical equipment as well as environmental exposure. Another study in Indonesia argues that contamination of birth attendant tools, possibly caused by poor cleaning equipment, increases the risk of infecting the newborn (38).

Beyond WASH-FIT-related variables, the regression found a significant correlation between hospital class and the proportion of maternal fatalities related to sepsis. The association between hospital class and the decrease in the proportion of maternal death rates could be attributable to the availability of more beds, services, human resources, buildings, and infrastructure as hospital class levels rise, or a timely referral system (39).

Only “adequate” environmental cleaning service is significantly and positively associated with patient satisfaction. In the Rifaskes dataset, all variables related to environmental cleaning services are about the availability of environmental cleaning procedures. One may see an indirect association between the environmental cleaning procedure and patient satisfaction (40, 41), e.g., the procedure makes the cleaning officers work properly, which results in a clean environment and then leads to high patient satisfaction. However, further research is needed to confirm our argument.

### 4.5 Study implications

Based on our analysis, we suggest that “good” environmental management and hygiene services help decrease maternal mortality from sepsis. Furthermore, by increasing environmental cleaning service facilities, the satisfaction of patients can be raised. Furthermore, waste management in HFCs should be the top priority in Indonesia.

From 2012 to 2018, the number of hospitals increased by 5.2% on average, with a total of 2,773 hospitals (42). According to the 2018 Central Statistics Agency data, 44.7% of medical waste generated each day still cannot be processed (43). Furthermore, multiple discrepancies were identified between the government’s waste management regulations and reality, including pressure or compaction of garbage using feet (44), waste bags that experienced leaks not being treated with double plastic bags, and abandonment in utilizing personal protective equipment (PPE) (45). It is also known that there are still personnel and employees who neglect the regulations for safe handling, storage, transportation, and disposal of hospital waste (46). This situation requires appropriate medical waste management to avoid environmental pollution, disease transmission, and occupational accidents (47).

Another significant factor to consider is efforts to enhance waste management in health facilities, which includes not only sorting and disposal but also reduction and sterilization so that waste output is decreased and does not harm the environment. Furthermore, sufficient budget for the health program and cross-sector coordination, e.g., between hospital, province or district level health agencies and environmental agencies, may enhance the improvement of waste management in hospital (48).

### 4.6 Research limitations

This study has some limitations. First, it is unknown whether patient satisfaction ratings are based just on WASH services or on satisfaction with hospital services in general, which include medical services, administrative services, and other supporting services. Second, the sanitation indicator can only identify the existence of separate toilets for workers and visitors, but the availability of bathrooms for visitors with disabilities and the amenities and also the level of safely managed sanitation services cannot be determined. Third, we can only use the data to assess the facilities for WASH services, but cannot assess the level of compliance for WASH services, such as hand-washing and environmental cleaning protocols. Fourth, the low percentage variance explained in the regression on patient satisfaction suggests that WASH-FIT indicators are not strongly correlated with patient satisfaction. Fifth, as mentioned previously, some findings and arguments still need to be clarified further. Sixth, our analysis using limited Rifaskes data may not fully explain the relationship between WASH-FIT indicators and maternal mortality and patient satisfaction in Indonesia. Finally, since we relied on secondary data, we have no control over the data collection process, data input, and what is contained in the dataset.

We also highlight the limited information available to fully measure the conditions of WASH-FIT indicators. Limited information also hinders us from fully analyzing the association between WASH-FIT indicators and maternal mortality and patient satisfaction. We suggest that more information can be collected in the next Rifaskes study. The Indonesia Ministry of Health can add some variables recommended by the WHO (1).

## 5 Conclusion

The WASH-FIT indicators were used to assess and monitor the status of WASH facilities in healthcare institutions in Indonesia. We categorized the six WASH-FIT services into three levels: poor, adequate, and good. The availability of WASH service facilities in hospitals in Indonesia was generally good, but waste management service still needs improvement. This suggests improvement in waste management is necessary for almost all hospitals in Indonesia. Variables that were significantly related to maternal mortality due to sepsis were adequate sanitation service, adequate and good levels of hand hygiene, environmental hygiene, and management services. Additionally, adequate environmental cleaning service was the only WASH-FIT indicator that was significantly associated with patient satisfaction. Finally, cross-sector collaboration was required to improve WASH services in hospitals, not just in terms of facilities, but also in WASH daily practices and behavior of all hospital workers and attendants.

## Data availability statement

The data analyzed in this study is subject to the following licenses/restrictions: the data analyzed in this study was obtained from Health Development Policy Agency (BKPK in Bahasa), Ministry of Health of Indonesia. Information and requests to access these datasets should be directed through this website: http://www.badankebijakan.kemkes.go.id/layanan-permintaan-data. Requests to access these datasets should be directed to BKPK, datin.bkpk@kemkes.go.id.

## Author contributions

RP: Data curation, Formal analysis, Investigation, Methodology, Resources, Software, Validation, Visualization, Writing – original draft, Writing – review & editing. DD: Conceptualization, Funding acquisition, Methodology, Project administration, Supervision, Validation, Writing – review & editing. FH: Conceptualization, Methodology, Supervision, Validation, Writing – review & editing.
