# Supplementary material for: Analysis of water, sanitation, and hygiene facilities using the WASH-FIT approach and its relation to patient satisfaction and maternal mortality at hospitals in Indonesia
Source: Front Public Health. 2024 Feb 1;12:1322470. doi: 10.3389/fpubh.2024.1322470 (PMC10867246; doi:10.3389/fpubh.2024.1322470)
Supplement: Supplementary file 1 [file Table_1.DOCX]

Supplementary Material

Table 1. Comparison of WASH Criteria based on WHO (WASH-FIT) and Rifaskes

| No. | Indicator | Criteria based on WASH-FIT | Criteria based on Rifaskes |
| --- | --- | --- | --- |
| Water Service | | | |
| 1 | Essential Indicators | Improved water supply piped into the facility or on premises and available | Availability of clean water sources: PAM |
| 2 |  | Water services available at all times and of sufficient quantity for all uses | Availability of clean water for 24 hours  Adequate availability of clean water |
| 3 |  | A reliable drinking-water station is present and accessible for staff, patients and carers at all times and in all locations/wards | Adequacy of clean water in the emergency room |
| 4 |  | Drinking-water is safely stored in a clean bucket/tank with cover and tap |  |
| 5 | Advanced Indicators | Sanitary inspection risk score (using sanitary inspection form 3) |  |
| 6 |  | All endpoints (i.e., taps) are connected to an available and functioning water supply |  |
| 7 |  | Water services available throughout  the year (i.e., not affected by seasonality, climate change-related extreme events or other constraints) |  |
| 8 |  | Water storage is sufficient to meet the needs of the facility for two days | Availability of a water reservoir (storage) |
| 9 |  | Water is treated and collected for drinking with a proven technology that meets WHO performance standards |  |
| 10 |  | Drinking water has appropriate chlorine residual (0.2 mg/L or 0.5 mg/L in emergencies) or 0 E. coli/100 ml and is not turbid |  |
| 11 |  | The facility water supply is regulated according to national water quality standards (mark not applicable if no standards exist) |  |
| 12 |  | Energy is available for heating water (mark if not applicable) |  |
| 13 |  | Energy is available for pumping water (mark if not applicable) |  |
| 14 |  | At least one shower or bathing area is available per 40 patients in inpatient settings and is functioning and accessible |  |
| Sanitatation Services | | | |
| 1 | Essential Indicators | Number of available and usable toilets or improved latrines for patients | Availability of outpatient toilets |
| 2 |  | Toilets or improved latrines clearly separated for staff and patients | Availability of staff toilets (in the emergency room) |
| 3 |  | Toilets or improved latrines clearly separated for male and female |  |
| 4 |  | At least one toilet or improved latrine provides the means to manage menstrual hygiene needs |  |
| 5 |  | At least one toilet meets the needs of people with reduced mobility |  |
| 6 |  | Functioning hand hygiene stations within 5 m of latrines |  |
| 7 |  |  | Availability of visitor toilets (in the emergency room) |
| 8 | Advanced Indicators | Record of cleaning toilets visible and signed by the cleaners each day |  |
| 9 |  | Wastewater is safely managed through use of on-site treatment (i.e. septic tank followed by drainage pit) or sent to a functioning sewer system | Availability of Wastewater Treatment Plant (WTP) |
| 10 |  |  | Availability of wastewater treatment permit |
| 11 |  | Greywater (i.e. rainwater or washwater) drainage system is in place that diverts water away from the facility (i.e. no standing water) and also protects nearby households |  |
| 12 |  | Latrines are adequately lit, including at night |  |
| Waste Management Service | | | |
| 1 | Essential Indicators | A trained person is responsible for the management of healthcare waste in the health care facility | Availability of a separate Hospital Waste Management Unit/Section/Installation |
| 2 |  | Functional waste collection containers in close proximity to all waste generation points for:  • non-infectious (general) waste  • infectious waste  • sharps waste | Sorting of medical solid waste  Sorting method used |
| 3 |  | Waste is correctly segregated at all waste generation points |  |
| 4 |  | Functional burial pit/fenced waste dump or municipal pick-up available for disposal of non-infectious (nonhazardous/general  waste) | Methods of infection control carried out in hospitals  pedal bin |
| 5 |  | Incinerator or alternative treatment technology for the treatment of infectious and sharp waste is functional and of a su­cient capacity | Methods of treatment of solid medical waste (treatment options)  • Incineration with incinerators  • Using an autoclave  • Using a microwave  • Buried in the ground with encapsulation techniques  • Disinfect with disinfectant  • Burned  • Treatment of solid medical waste with an incinerator is carried out at this health facility |
| 6 |  | Sucient energy available for incineration or alternative treatment technologies (mark if not applicable) |  |
| 7 |  |  | Safety box ownership |
| 8 |  |  | Needle destroyer ownership |
| 9 | Advanced Indicators | Hazardous and non-hazardous waste are stored separately before being treated/disposed of or moved off-site | Storage of radioactive waste in separate containers  Storage of cytotoxic waste in separate containers  Storage of chemical and pharmaceutical waste in separate containers |
| 10 |  | All infectious waste is stored in a protected area before treatment, for no longer than the default and safe time | Availability of temporary storage area for toxic and hazardous waste (B3)  Methods of infection control carried out in hospitals  safety box  auto disposable syringe |
| 11 |  | Anatomical/pathological waste is put in a dedicated pathological waste/placenta pit, burnt in a crematory or buried in a cemetery (mark if not applicable) |  |
| 12 |  | Dedicated ash pits available for disposal of incineration ash (mark if not applicable) |  |
| 13 |  | Protocol or standard operating procedure (SOP) for safe management of health care waste clearly visible and legible | Availability of SOP for waste disposal |
| 14 |  | Appropriate protective equipment for all staff in charge of waste treatment and disposal | Methods of infection control carried out in hospitals: Disposable latex gloves |
| Hand Hygiene Services | | | |
| 1 | Essential Indicators | Functioning hand hygiene stations are available at all points of care | Methods of infection control carried out in hospitals  • Clean running water  • Alcohol hand rub |
| 2 |  | Hand hygiene promotion materials clearly visible and understandable at key places | Installation of health banners/banners/posters |
| 3 |  |  |  |
| 4 | Advanced Indicators | Functioning hand hygiene stations are available in service areas |  |
| 5 |  | Functioning hand hygiene stations available in the waste disposal area |  |
| 6 |  | Hand hygiene compliance activities are undertaken regularly |  |
| Environmental Cleaning Services | | | |
| 1 | Essential Indicators | The exterior of the facility is well-fenced, and kept generally clean (free from solid waste, stagnant water, no animal and human faeces in or around the facility premises, etc.) |  |
| 2 |  | General lighting su­ciently powered and adequate to ensure safe provision of health care including at night (mark if not applicable) |  |
| 3 |  | Floors and horizontal work surfaces appear clean |  |
| 4 |  | Appropriate and well maintained materials for cleaning (i.e. detergent, mops, buckets, etc.) are available |  |
| 5 |  | At least two pairs of household cleaning gloves and one pair of overalls or apron and boots in a good state, for each cleaning and waste disposal staff Member | Existence of SOPs for the usage of personal protective equipment (PPE) |
| 6 |  | At least one member of staff can demonstrate the correct procedures for cleaning and disinfection and apply them as required to maintain clean and safe rooms | Availability of procedures for handling toxic and hazardous waste contamination |
| 7 |  | Beds have insecticide-treated nets to protect patients from mosquito-borne diseases |  |
| 8 | Advanced Indicators | A mechanism exists to track the supply of IPC-related materials (such as gloves and protective equipment) to identify stock-outs |  |
| 9 |  | Record of cleaning visible and signed by the cleaners each day |  |
| 10 |  | Laundry facilities are available to wash linen from patient beds between each patient | Availability of laundry/laundry services |
| 11 |  | The facility has sufficient natural ventilation and where the climate allows, large opening windows, skylights and other vents to optimize natural ventilation |  |
| 12 |  | Kitchen stores and prepared food is  protected from flies, other insects or rats |  |
| 13 |  | Beds for patients should be separated by 2.5 m from the centre of one bed to the next and each bed should have only one patient |  |
| Management Services | | | |
| 1 | Essential Indicators | WASH FIT or other quality improvement/management plan for the facility is in place, implemented and regularly monitored | Availability of hospital strategic plan documents  Organizing in carrying out the strategic plan  Availability of implementation documents  Implementation of evaluation monitoring |
| 2 |  | An annual planned budget for the facility is available and includes funding for WASH infrastructure, services, personnel and the continuous procurement of WASH items (hand hygiene products, minor supplies to repair pipes, toilets, etc.) which is su­cient to meet the needs of the facility | Availability of budget for the implementation of health promotion activities in hospitals |
| 3 |  | An up-to-date diagram of the facility management structure is clearly visible and legible | Hospital organizational structure |
| 4 |  | Adequate cleaners and WASH maintenance staff are available |  |
| 5 | Advanced Indicators | A protocol for operation and maintenance, including procurement of WASH supplies is visible, legible and implemented | Hospital occupational health and safety program (policy)  Availability of standard infection prevention precautions guidelines |
| 6 |  | Regular ward-based audits are undertaken to assess the availability of handrub, soap, single-use towels and other hand hygiene resources |  |
| 7 |  | New healthcare personnel receive IPC training as part of their orientation programme |  |
| 8 |  | Health care staff are trained on WASH/IPC each year | Staff education and training (development) program in occupational safety, fire hazard and disaster in 2018 |
| 9 |  | The facility has a dedicated WASH or IPC  focal person | Availability of Nosocomial Infection Control Committee or Infection Prevention and Control (IPC) |
| 10 |  | All staff have a job description written clearly and legibly, including WASH-related responsibilities and are regularly appraised on their performance |  |
| 11 |  | High performing staff are recognized and rewarded and those that do not perform are dealt with accordingly |  |
